# Early Neuropathy as a Predictor of Subclinical Diabetic Nephropathy in Well-Controlled Type 2 Diabetic Patients: A Cross-Sectional Study

**DOI:** 10.1155/jdr/3736035

**Published:** 2025-11-10

**Authors:** Sherif Mohamed Zaki, Dina El Karsh, Ghosoun Anas Moallem, Abdulrahman Hatem Adel Sembawa, Moayad Saeed Omar Alsubhani, Abdulaziz Mahmoud Wahbi Sindi, Mohammed Khalid Omar Alafif, Faisal Waleed Abdullah Mulla

**Affiliations:** ^1^Physiological Sciences Department, MBBS program, Fakeeh College for Medical Sciences, Jeddah, Saudi Arabia; ^2^Department of Family Medicine, Dr. Soliman Fakeeh Hospital, Jeddah, Saudi Arabia; ^3^Bachelor of Medicine, Bachelor of Surgery Program, Fakeeh College for Medical Sciences, Jeddah, Saudi Arabia

**Keywords:** albuminuria, diabetic nephropathy, diabetic neuropathy, early kidney disease, Toronto clinical score, Type 2 diabetes

## Abstract

**Background:**

Diabetic neuropathy (DN) and nephropathy (DKD) are prevalent microvascular complications in Type 2 diabetes mellitus (T2DM), often evolving silently. Detecting early nephropathy remains a clinical challenge, especially in patients with preserved renal function.

**Objective:**

The objective was to determine whether the Toronto Clinical Scoring System (TCS) for diabetic neuropathy can predict early nephropathy (albuminuria) in people with well-controlled T2DM who have a normal eGFR.

**Methods:**

We conducted a cross-sectional study with 122 T2DM patients (HbA1c < 7%, eGFR > 90) to look for peripheral neuropathy using TCS and nephropathy using the urinary albumin-to-creatinine ratio (UACR). Patients were classified based on the presence of albuminuria (UACR ≥ 30 mg/g). Statistical analyses included *t*-tests, chi-square tests, Spearman correlation, and logistic regression.

**Results:**

Patients with diabetic nephropathy or neuropathy were significantly older and exhibited higher systolic blood pressure and albuminuria. A clear stepwise increase in albuminuria was observed with rising neuropathy severity, with nephropathy prevalence ranging from 42% in patients without neuropathy to 72% in those with severe neuropathy. A significant positive correlation between TCS and UACR (*ρ* = 0.29, *p* = 0.0012) supports a progressive link between nerve and kidney involvement.

**Conclusion:**

Clinical diabetic neuropathy is significantly associated with early nephropathy in well-controlled T2DM patients. Routine neuropathy assessment may serve as a simple, cost-effective predictor of subclinical renal damage. Future prospective studies should investigate whether early intervention in patients with neuropathy can attenuate or delay renal injury and whether this predictive link holds true across diverse ethnic and age groups.

## 1. Introduction

Diabetes mellitus is highly prevalent in Saudi Arabia, leading to a significant burden of chronic complications. Saudi Arabia ranks among the top countries globally for diabetes prevalence, with roughly 17%–25% of adults affected [1]. Diabetic nephropathy (DN) and diabetic peripheral neuropathy (DPN) are two major microvascular complications of diabetes mellitus [2]. They often develop insidiously and impact quality of life and mortality [3]. DN and DPN are prevalent and interlinked complications in Saudi Arabia. Prevalence rates are alarmingly high—roughly one-quarter to one-third of Saudi diabetics develop DN [4], and a similar proportion suffer DPN [5].

Poor glycemic control and longer diabetes duration are the strongest predictors for developing microvascular complications. Patients with a longer history of diabetes have far higher odds of nephropathy or neuropathy [6, 7]. In one Saudi study, every 5-year increase in diabetes duration was associated with 2.5-fold higher odds of peripheral neuropathy [6]. Furthermore, nephropathy was much more common in people who had diabetes for more than 10 years. Saudi registry data showed that people with nephropathy had had diabetes for an average of 18.8 years, compared to 12.8 years for people without nephropathy (*p* < 0.0001) [7]. Hyperglycemia (high HbA1c) is a critical risk factor—chronically elevated blood glucose damages small vessels supplying nerves and kidneys. Over 60% of Saudi patients with DN have HbA1c > 8%—a significantly higher proportion than in patients without nephropathy [7].

While hyperglycemia is the key pathogenic driver, recent evidence suggests that these complications can develop even in patients with well-controlled HbA1c levels [8]. This raises the need for identifying early markers that may predict end-organ damage before classical indicators such as reduced eGFR become apparent [9]. Given that neuropathy may precede or coexist with nephropathy [10], the presence of diabetic neuropathy could serve as an early clinical predictor of renal involvement, particularly albuminuria.

Although DN and DPN may occur independently, both share common microvascular and metabolic pathways, including endothelial dysfunction, advanced glycation end products, and chronic low-grade inflammation [11, 12]. This overlap provides a strong biological rationale for investigating whether one complication may serve as a clinical warning sign for the other. Importantly, neuropathy is often symptomatic (pain and numbness), whereas nephropathy evolves silently until advanced stages [13]. Therefore, the presence of neuropathy could offer an early clinical cue for otherwise subclinical renal damage. While our cross-sectional design cannot establish causality, our study hypothesizes that neuropathy severity, assessed by the Toronto Clinical Scoring System (TCS), may predict early nephropathy risk in well-controlled Type 2 diabetic patients [10, 14, 15].

The aim of our study was to assess whether the presence and severity of diabetic neuropathy can predict the presence of early nephropathy (i.e., micro- or macroalbuminuria) in patients with well-controlled Type 2 diabetes and whose kidney function is still good. Our hypothesis was that among Type 2 diabetic patients with HbA1c < 7% and eGFR > 90 mL/min/1.73 m^2^, the presence of diabetic neuropathy is associated with an increased prevalence of albuminuria (UACR ≥ 30 mg/g).

## 2. Materials and Methods

### 2.1. Study Design and Setting

This cross-sectional study was conducted at Dr. Soliman Fakeeh Hospital, Jeddah, Saudi Arabia, over a 12-month period from January to December 2024. Ethical approval was obtained from the Institutional Review Board of Dr. Soliman Fakeeh Hospital (652/IRB/2024). In line with the Declaration of Helsinki's tenets, written informed consent was acquired from each participant.

### 2.2. Study Population

We collected 122 patients with T2DM from the family medicine clinic.

Inclusion criteria were adults aged 35–70 years, diagnosed with T2DM, HbA1c < 7% within the last 3 months, and estimated glomerular filtration rate (eGFR) ≥ 90 mL/min/1.73 m^2^. This threshold was chosen to avoid including patients with preexisting chronic kidney disease and to focus specifically on subclinical nephropathy in individuals with preserved renal function.

Patients were excluded if they had a history of chronic kidney disease or eGFR < 90 mL/min/1.73 m^2^, macrovascular complications (e.g., cerebrovascular accident and ischemic heart disease), use of nephrotoxic medications or ACE inhibitors at the time of recruitment, or other potential causes of peripheral neuropathy (e.g., vitamin B12 deficiency, hypothyroidism, chronic alcohol use, and neurotoxic medications).

### 2.3. Clinical and Laboratory Evaluation

Demographic and clinical data collected included age, gender, BMI, duration of diabetes, and smoking status. As part of the laboratory tests performed, HbA1c, fasting plasma glucose, serum creatinine, eGFR (CKD-EPI), UACR from a morning spot urine sample, and a lipid profile (total cholesterol, LDL, HDL, and triglycerides) were all measured.

### 2.4. Assessment of Diabetic Neuropathy

Peripheral neuropathy was evaluated using TCS. TCS is a practical tool that can be used in outpatient clinics to detect neuropathy early and categorize its severity. This system is a 19-point composite score based on neuropathic symptoms and physical examination findings [16].

### 2.5. TCS Evaluates

#### 2.5.1. Symptoms (0–6 Points)

Presence of six symptom categories—foot pain, foot numbness, tingling, weakness, ataxia, and upper limb symptoms—each scored 1 if present, 0 if absent.

#### 2.5.2. Sensory Tests (0–5 Points)

Five modalities tested on the great toe—pinprick, temperature, light touch, vibration, and position sense—each scored 1 if abnormal or reduced, 0 if normal.

#### 2.5.3. Reflexes (0–4 Points)

Deep tendon reflexes at the knees and ankles, graded 0 if normal, 1 if reduced, and 2 if absent (max 2 points for each of knee and ankle reflexes).

Scores range from 0 (normal) to 19 (maximal neuropathy). Neuropathy severity was categorized as 0–5: *no neuropathy*; 6–8: *mild neuropathy*; 9–11: *moderate neuropathy*; and ≥12: *severe neuropathy* [16].

The TCS was selected because it is a validated, reliable, and cost-effective clinical tool that is widely applied in both outpatient and epidemiological research to detect and grade neuropathy.

### 2.6. Assessment of DN

UACR was used to check for kidney damage. Nephropathy was categorized as normoalbuminuric (UACR < 30 mg/g), microalbuminuria (UACR 30–300 mg/g), and macroalbuminuria (UACR > 300 mg/g) [12]. We divided the patients into the following groups: no nephropathy: UACR < 30 mg/g and early nephropathy: UACR ≥ 30 mg/g (including both micro- and macroalbuminuria).

### 2.7. Statistical Analysis

Data analysis was performed using SPSS Version 16. Continuous variables were expressed as mean ± standard deviation (SD) and categorical variables as frequencies and percentages.

Comparisons between groups (with vs. without nephropathy) were conducted using an independent *t*-test for continuous variables and a chi-square test for categorical variables.

We conducted a correlation analysis using the Spearman correlation between TCS and UACR levels. Spearman's *ρ* = 0.43, *p* = 0.042, indicating a moderate and statistically significant positive correlation between neuropathy severity and albuminuria.

We used a binary logistic regression analysis to look at the link between diabetic neuropathy (based on TCS) and early nephropathy (UACR ≥ 30 mg/g), considering factors like age, gender, length of diabetes, and lipid profile that might have changed the relationship. A *p* value < 0.05 was considered statistically significant.

## 3. Results

The mean BMI for the 122 well-controlled Type 2 diabetic patients was 31.62 ± 6.28, their HbA1c level was 6.22% ± 0.49%, their systolic blood pressure was 130.20 ± 16.17 mmHg, and their eGFR stayed at 91 mL/min/1.73 m^2^.

Table 1 presents the mean ± SD of the clinical and biochemical variables in patients with and without DN and diabetic neuropathy. Patients with DN showed higher mean age (64.0 vs. 61.9 years), systolic blood pressure (133.0 vs. 126.2 mmHg), and UACR (119.0 vs. 8.6 mg/g) compared to those without nephropathy.

**Figure 1:**
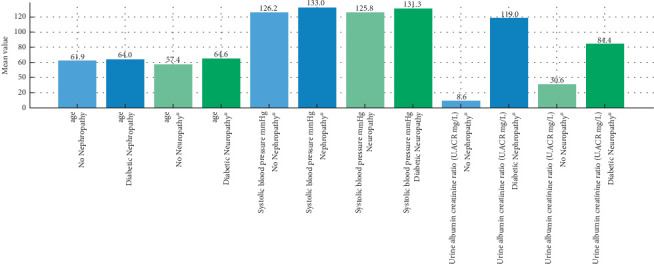
Significant differences in clinical variables among the nephropathy and neuropathy groups. Each pair of bars corresponds to a clinical variable, contrasting affected and unaffected patients. Asterisks (⁣^∗^) denote statistically significant differences (*p* < 0.05).

Furthermore, people with neuropathy were older (64.6 vs. 57.4 yrs), had higher systolic blood pressure (131.3 vs. 125.8 mmHg), and had higher UACR (84.4 vs. 30.6 mg/g) than those without neuropathy (Figure 1). No significant differences in BMI, HbA1c, or diastolic BP were observed.

As shown in Table 2, the chi-square analyses compared each categorical variable across nephropathy versus no nephropathy and neuropathy versus no neuropathy groups. The final row summarizes the overlap between the two complications. DN was significantly associated with hypertension, while diabetic neuropathy showed a significant association with the duration of diabetes. No significant associations were observed with sex, smoking status, or lipid parameters.

**Figure 2:**
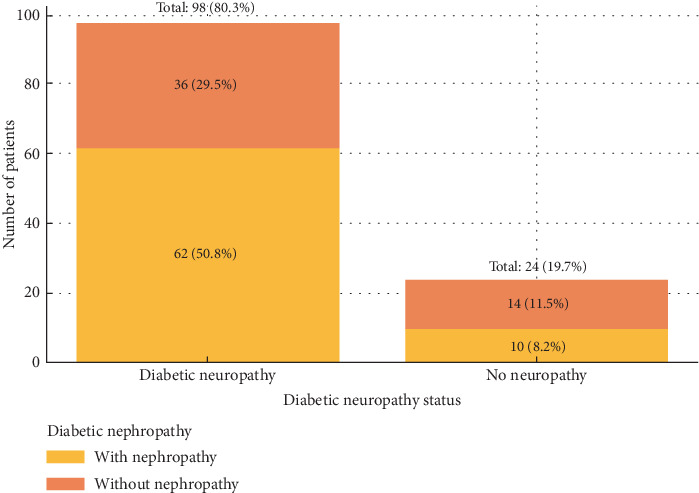
demonstrates the association between diabetic neuropathy and diabetic nephropathy, with bar segments representing the percentage of the total study population.

Among all participants, 62 patients (50.8%) had both diabetic neuropathy and nephropathy, while 36 patients (29.5%) had neuropathy without nephropathy. Just 14 patients (11.5%) had both complications, whereas 10 patients (8.2%) had nephropathy without neuropathy (Figure 2).

**Figure 3:**
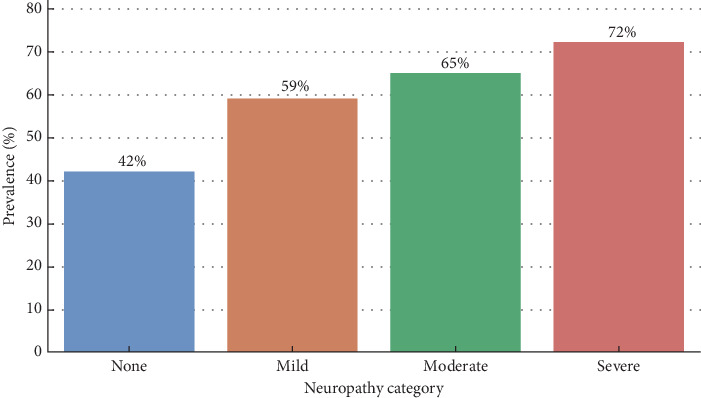
“Prevalence of nephropathy by neuropathy category.” This bar chart offers a visual overview of the progressive relationship between diabetic neuropathy and nephropathy.

Figure 3 shows a stepwise association between the grade of neuropathy and the extent of renal involvement. Progressive and significant increases in nephropathy prevalence were identified per neuropathy category. Nephropathy was present in 42% of patients without clinical neuropathy. This prevalence rose to 59% in patients with mild neuropathy (*n* = 27), 65% with moderate neuropathy (*n* = 34), and 72% in the severe neuropathy group (*n* = 29).

**Figure 4:**
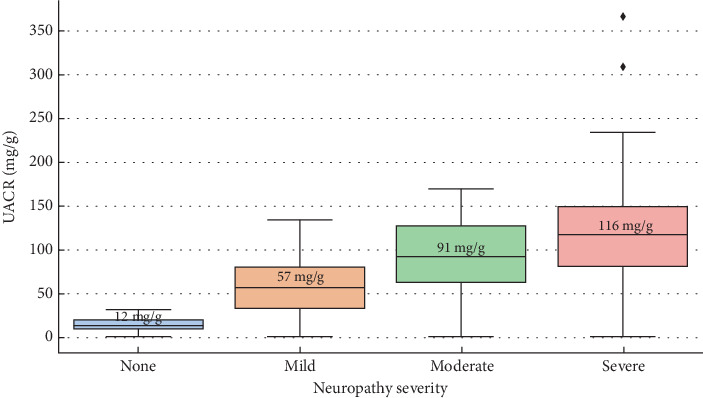
“UACR levels by neuropathy severity.” This boxplot visualizes the distribution of UACR across four neuropathy severity categories.

Figure 4 demonstrates how the UACR changes in four levels of neuropathy severity. Patients without neuropathy had the lowest median UACR, with a narrower interquartile range and fewer outliers. Patients with mild, moderate, and severe neuropathy presented increasingly higher median UACR levels.

**Figure 5:**
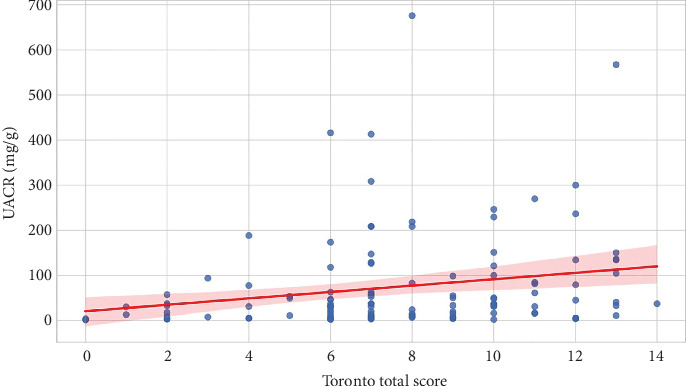
“Correlation between TCS and UACR.” This scatter plot demonstrates a positive linear relationship between TCS and UACR. Each dot represents an individual patient, with the red regression line and shaded confidence interval showing the trend.

A Spearman correlation revealed a positive linear relationship between TCS and UACR (*ρ* = 0.29, *p* = 0.0012), suggesting that patients with more severe neuropathy are more likely to have elevated levels of albuminuria (Figure 5).

## 4. Discussion

In this cross-sectional study of well-controlled Type 2 diabetic patients with preserved renal function, we found that peripheral neuropathy—as measured by TCS—is a significant predictor of early DN, evidenced by elevated UACR. Recent research suggests a bidirectional relationship between kidney disease and neuropathy, where markers of one condition may predict the development or progression of the other [17]. Urinary markers of glomerular and tubular damage can be used for early prediction of DPN [17]. Conversely, elevated UACR and decreased eGFR might be predictive factors of DPN [17]. Furthermore, neuropathic changes may serve as early indicators of silent renal injury in diabetes [18].

The positive correlation between neuropathy severity and albuminuria (*ρ* = 0.29, *p* = 0.0012) underlines the shared microvascular pathophysiology of both complications [13]. Chronic hyperglycemia, though well controlled in our cohort (mean HbA1c = 6.22% ± 0.49%), can induce endothelial dysfunction and microvascular damage within both neural and renal tissues [12, 19]. This conclusion is consistent with findings that DPN may occur earlier than DN in the progression of Type 2 diabetes mellitus [11].

Despite narrow variability in HbA1c, over half of our patients exhibited evidence of nephropathy or neuropathy, with 50.8% affected by both. This dissociation between glycemic control and complication onset is consistent with studies indicating that diabetic neuropathy can be present even in patients with good glycemic control [14], as there are many other factors involved, like dyslipidemia, hypertension, subclinical neuropathy at diagnosis, and genetic susceptibility [20, 21]. Additionally, the duration of diabetes and other metabolic factors (blood pressure, lipids, and obesity) contribute to neuropathy even when recent HbA1c is well controlled [22].

Hypertension emerged as a strong independent associate of nephropathy in our analysis, consistent with KDIGO guidelines that emphasize blood pressure as a critical modifiable determinant of renal prognosis in diabetes [23]. Notably, neuropathy in our study was significantly associated with the duration of diabetes—a well-established risk enhancer that amplifies vascular degeneration over time [24].

Our findings support the utility of routine neuropathy screening, not only for neurologic symptom management but also as a predictive tool for renal risk stratification. TCS is cost-effective and noninvasive [16]. It has been used as a bedside screening tool for DPN in resource-limited settings [16]. Recent studies have also translated and authorized the TCS in other languages (e.g., Spanish) and settings, confirming it as a strong clinical tool for DPN across populations [25]. TCS could be particularly valuable in primary care settings where advanced renal biomarkers may be inaccessible.

Moreover, the graded increase in albuminuria observed across ascending neuropathy severity levels suggests that neuropathy may not only be concurrent with nephropathy but may precede it. This hypothesis aligns with preceding work indicating that microvascular damage in peripheral nerves may occur before overt renal dysfunction becomes biochemically obvious [15].

## 5. Limitations and Conclusion

Our study has several limitations. First, neuropathy was assessed exclusively using the TCS. Although TCS is a validated, reliable, and cost-effective tool widely used in outpatient and epidemiological research, the absence of pathological confirmation such as intraepidermal nerve fiber (IENF) density or electrophysiological studies may reduce diagnostic certainty. These methods were not feasible in the current cross-sectional outpatient setting, but future longitudinal studies should integrate such objective assessments alongside TCS to strengthen diagnostic accuracy.

Second, the cross-sectional design prevents us from establishing temporal or causal relationships between neuropathy and nephropathy. Prospective cohort studies are required to confirm whether neuropathy precedes nephropathy and to clarify the directionality of this association.

Third, we did not include potential confounding variables such as inflammatory markers, oxidative stress indices, or detailed medication use (e.g., ACE inhibitors, ARBs, or neuroprotective agents). Future studies should incorporate these factors to provide a more comprehensive understanding of the relationship between neuropathy and nephropathy.

Fourth, although we restricted our cohort to patients with eGFR ≥ 90 to exclude chronic kidney disease, we acknowledge that glomerular hyperfiltration may occur in the early stages of DN and may not have been fully captured. Furthermore, age-related neurological changes could confound the assessment of diabetic neuropathy, and this should be considered when interpreting our findings.

In conclusion, our findings demonstrate that DPN, as measured by TCS, is significantly associated with early nephropathy in well-controlled Type 2 diabetic patients with preserved renal function. Routine neuropathy screening may therefore serve as a simple, accessible predictor of subclinical renal involvement. Future research should determine whether early detection and intervention in neuropathy can alter the trajectory of diabetic kidney disease and improve long-term outcomes.

## Figures and Tables

**Table 1:** Comparative analysis of clinical and biochemical variables across the nephropathy and neuropathy groups.

**Variable**	**Nephropathy**			**Neuropathy**		
	**No nephropathy** **(** **n** = 72**)**	**Nephropathy** **(** **n** = 50**)**	**p** ** value**	**No neuropathy** **(** **n** = 98**)**	**Neuropathy** **(** **n** = 24**)**	**p** ** value**
Age (years)	62 ± 9	64 ± 12	0.27	57 ± 10	65 ± 10	0.00⁣^∗^
BMI (kg/m^2^)	31 ± 5	32 ± 7	0.49	33 ± 5	31 ± 6	0.15
HbA1c (%)	6 ± 0.49	6 ± 0.49	0.81	6 ± 0.56	6 ± 0.47	0.30
Diastolic BP (mmHg)	76 ± 9	76 ± 10	0.75	76 ± 9	76 ± 10	0.86
Systolic BP (mmHg)	126 ± 14	133 ± 17	0.02∗	126 ± 16	131 ± 16	0.14
TG (mg/dL)	120 ± 79	138 ± 108	0.29	126 ± 76	132 ± 102	0.75
HDL (mg/dL)	48 ± 10	46 ± 11	0.29	47 ± 10	47 ± 11	0.87
LDL (mg/dL)	101 ± 82	85 ± 34	0.20	90 ± 28	92 ± 64	0.83
Hb (g/dL)	14 ± 1	14 ± 2	0.66	14 ± 2	14 ± 2	0.46
UACR (mg/L)	9 ± 6	119 ± 124	0.00⁣^∗^	31 ± 42	84 ± 118	0.00⁣^∗^
eGFR (mL/min/1.73 m^2^)	101 ± 6	102 ± 9	0.68	101 ± 7	102 ± 8	0.42

*Note:* Significance was determined using independent samples *t*-tests.

Abbreviations: BMI: body mass index; BP: blood pressure; eGFR: estimated glomerular filtration rate; Hb: hemoglobin; HbA1c: hemoglobin A1c; HDL: high-density lipoprotein; LDL: low-density lipoprotein; *p*: *p* value; SD: standard deviation; TGs: triglycerides; UACR: urinary albumin-to-creatinine ratio; Yrs: years.

⁣^∗^Statistically significant associations at *p* < 0.05.

**Table 2:** Nephropathy–neuropathy association in diabetic patients.

**Variable**	**Category**	**Nephropathy**							**Neuropathy**						
		**No nephropathy**		**Nephropathy**		**Total**		**p** ** value**	**No neuropathy**		**Neuropathy**		**Total**		**p** ** value**
		**Count**	**%**	**Count**	**%**	**Count**	**%**		**Count**	**%**	**Count**	**%**	**Count**	**%**	
Sex	Female	34	27.9	19	15.6	53	43.5	0.41	41	33.6	12	9.8	53	43.4	0.62
	Male	38	31.1	31	25.4	69	56.5		57	46.7	12	9.8	69	56.5	
Smoking status	Nonsmoking	43	35.2	31	25.4	74	60.6	0.95	60	49.2	14	11.5	74	60.7	0.98
	Smoking	29	23.8	19	15.6	48	39.4		38	31.1	10	8.2	48	39.3	
Duration of diabetes	Less than 10 years	30	24.6	20	16.4	50	41	1	35	28.7	15	12.3	50	41	0.03⁣^∗^
	More than 10 years	42	34.4	30	24.6	72	59		63	51.6	9	7.4	72	59	
Blood pressure	Hypertension	49	40.2	22	18	71	58.2	0.01⁣^∗^	59	48.4	12	9.8	71	58.2	0.5
	Normal	23	18.9	28	23	51	41.9		39	32	12	9.8	51	41.8	
TG level	Hypertriglyceridemia	17	13.9	9	7.4	26	21.3	0.6	24	19.7	2	1.6	26	21.3	0.15
	Normal	55	45.1	41	33.6	96	78.7		74	60.7	22	18	96	78.7	
HDL level	Low	19	15.6	9	7.4	28	23	0.39	24	19.7	4	3.3	28	23	0.59
	Normal	53	43.4	41	33.6	94	77		74	60.7	20	16.4	94	77.1	
LDL level	High	7	5.7	9	7.4	16	13.1	0.29	12	9.8	4	3.3	16	13.1	0.81
	Normal	65	53.3	41	33.6	106	86.9		86	70.5	20	16.4	106	86.9	
Anemic status	Anemia	20	16.4	8	6.6	28	23	0.19	23	18.9	5	4.1	28	23	1
	Normal	52	42.6	42	34.4	94	77		75	61.5	19	15.6	94	77.1	
Serum creatinine level	Elevated	15	12.3	7	5.7	22	18	0.47	21	17.2	1	0.8	22	18	0.09
	Normal	57	46.7	43	35.2	100	81.9		77	63.1	23	18.9	100	82	
Diabetic retinopathy	Diabetic retinopathy	7	5.7	10	8.2	17	13.9	0.18	13	10.7	4	3.3	17	14	0.92
	No retinopathy	65	53.3	40	32.8	105	86.1		85	69.7	20	16.4	105	86.1	
Nephropathy vs neuropathy⁣^∗∗^		14	11.5	36	29.5	50	41	0.09	10	8.2	62	50.8	72	59	0.09

*Note: p* values represent chi-square comparisons between (a) patients with versus without nephropathy and (b) patients with versus without neuropathy for each categorical variable.

⁣^∗^Statistically significant associations at *p* < 0.05.

⁣^∗∗^The last row (“nephropathy vs. neuropathy”) summarizes the overlap between the two complications, showing patients with neuropathy only, nephropathy only, both, or neither.

## Data Availability

The data supporting the findings of this study are available from the corresponding author upon reasonable request. All data were anonymized to ensure participant confidentiality in accordance with institutional ethical standards.
